# Prolonged low-dose methylprednisolone treatment is highly effective in reducing duration of mechanical ventilation and mortality in patients with ARDS

**DOI:** 10.1186/s40560-018-0321-9

**Published:** 2018-08-24

**Authors:** Gianfranco Umberto Meduri, Reed A. C. Siemieniuk, Rachel A. Ness, Samuel J. Seyler

**Affiliations:** 1 Department of Medicine, Division of Pulmonary, Critical Care and Sleep Medicine, Memphis Veterans Affairs Medical Center (111), 1030 Jefferson Avenue, Suite room #CW444, Memphis, TN 38104 USA; 2 Department of Health Research Methods, Evidence, and Impact, McMaster University, Hamilton, Ontario Canada; 3 Department of Medicine, University of Toronto, Toronto, Ontario Canada; 4 Department of Pharmacy, Memphis Veterans Affairs Medical Center, Memphis, TN USA; 5 Department of Medicine and Pediatrics, University of Tennessee Health Science Center, Memphis, TN USA

**Keywords:** Adult respiratory distress syndrome, Glucocorticoid treatment, Methylprednisolone, Dosage, Duration of treatment, Duration of mechanical ventilation, Tapering, Reconstituted systemic inflammation, Survival

## Abstract

An updated meta-analysis incorporating nine randomized trials (*n* = 816) investigating low-to-moderate dose prolonged glucocorticoid treatment in acute respiratory distress syndrome (ARDS) show moderate-to-high quality evidence that glucocorticoid therapy is safe and reduces (i) time to endotracheal extubation, (ii) duration of hospitalization, and (iii) mortality (number to treat to save one life = 7), and increases the number of days free from (i) mechanical ventilation, (ii) intensive care unit stay, and (iii) hospitalization. Recent guideline suggests administering methylprednisolone in patients with early moderate-to-severe (1 mg/kg/day) and late persistent (2 mg/kg/day) ARDS (conditional recommendation based on moderate quality of evidence).

## Background

The English version of the clinical practice guidelines for the management of adult patients with ARDS (Japanese version publication July 2016) was recently published in the *Journal of Intensive Care* [1]. The recommendations are based on only five heterogeneous randomized controlled trials (RCTs) published before 2008. Four RCTs investigated methylprednisolone: one trial (1987) [2] administered 120 mg/kg over 24 h and the other (1998–2007) used 1 mg/kg/day in patients with early ARDS [3] or 2 mg/kg/day in patients with late ARDS [4, 5] over 4 weeks. The other trial investigated hydrocortisone and fludrocortisone over 7 days in patients with ARDS and vasopressor-dependent septic shock. The clinical practice guideline development (CPGD) committee concluded that there was no significant reduction in mortality, no increased risk for infection, and an increase in mechanical ventilation-free days (MVFD). The CPGD committee concluded that the overall quality of evidence across outcomes was “moderate” to suggest (GRADE 2B) the use of methylprednisolone in a dosage of 1 to 2 mg/kg/day. In addition, the CPGD committee referenced a 2014 domestic survey reporting the common practice among Japanese doctors of administering 500–1000 mg/day (pulse dose) of methylprednisolone to patients with ARDS. We wish to present a brief update of the literature and updated guidelines that might be of practical importance to clinicians.

## Main body

Recent experimental [6] and clinical research [7, 8] suggest that pulse dose methylprednisolone may not be beneficial in ARDS. In an experimental study, rats with lipopolysaccharide-induced acute lung injury were exposed to graded concentrations of methylprednisolone (3 mg, 30 mg, 180 mg) for up to 14 days [6]. Serial BAL and lung histology demonstrated more significant improvements at 12 h in the higher dose group. However, by day 7, the high-dose group had partial loss of early laboratory improvements and significantly worsen pathological scores, while the lower dose group achieved continued improvement in both pathological and laboratory variables [6]. Similarly, data from two recent retrospective studies suggests that pulse dose steroids may be harmful [7, 8]. For example, in a retrospective comparison with low-dose methylprednisolone (0.5–1 mg/kg/day; *n* = 165), high-dose methylprednisolone (1000 mg/day for 3 days followed by 2 mg/kg/day) was associated with higher 60-day mortality and a 10-day reduction in ventilator-free days by day 28 [7].

A multi-specialty task force of international experts assembled by the Society of Critical Care Medicine and the European Society of Intensive Care Medicine recently published the updated guidelines for the diagnosis and treatment of Critical Illness Related Corticosteroid Insufficiency (CIRCI) [9]. In a separate document [10], the task force reviewed clinical and experimental evidence on the central role played by CIRCI in the pathobiology of ARDS and how increasing glucocorticoid receptorα (GRα) activation with quantitatively adequate and prolonged glucocorticoid supplementation can reverse CIRCI and accelerate resolution of pulmonary and systemic inflammation. The impact of methylprednisolone treatment on GRα number and function in patients with ARDS was also reviewed [11, 12]. For their recommendations, the task force relied mostly on a recent systematic review of RCTs investigating prolonged (7 days or greater) glucocorticoid treatment in ARDS [13]. This systematic review included a primary individual patient data meta-analysis (IPDMA) of four RCTs investigating methylprednisolone treatment (*n* = 322) [3–5, 14], and an aggregate data meta-analysis incorporating four additional RCTs [15–18] investigating hydrocortisone treatment in early ARDS (*n* = 297). There were substantial differences in the treatment protocol design. Data are presented as methylprednisolone vs. hydrocortisone: an initial bolus was used in 4 [3–5, 14] vs. 2 [15, 17]; duration of treatment was 24 to 32 days vs 7 days, and slow tapering of study drug was implemented in 3 [3, 4, 14] vs. none.

**Fig. 1:**
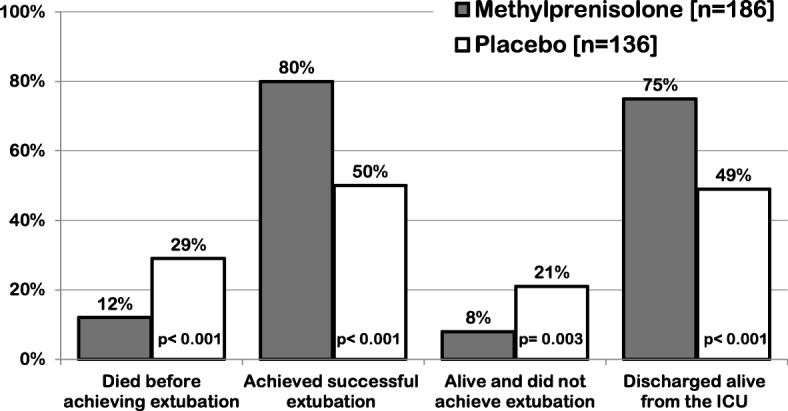
Individual patient data from four randomized trials investigating prolonged methylprednisolone treatment in ARDS [13]: outcome related to achieving extubation and intensive care unit discharge by day 28

Individual patient data meta-analyses allow for time-to-event analyses and examination of new outcomes not previously reported (e.g., MV-free days and impact of tapering). By study day 28 (Fig. 1), fewer patients in the methylprednisolone group died before extubation (12% vs. 29%; *p* < 0.001) and more patients achieved extubation (80% vs. 50%; *p* < 0.001) and were discharged alive from the intensive care unit (75% vs 49%; *p* < 0.001). In the methylprednisolone group, time to extubation was shorter (hazard ratio 2.59, 95% CI 1.95–3.43, *p* < 0.001) and hospital mortality was lower (20% vs. 33%; *p* = 0.006). The results were similar in both the aggregate (eight studies) and individual patient meta-analyses (four studies). In addition, prolonged methylprednisolone treatment was associated with (i) increased MV-free days (13.3 ± 11.8 vs. 7.6 ± 5.7; *p* < 0.001), ICU-free days (10.8 ± 0.71 vs. 6.4 ± 0.85; *p* < 0.001), and hospital-free days to day 28 (7.0 ± 0.57 vs. 3.8 ± 0.68; *p* < 0.001), and a reduction in development of shock (3% vs. 15%; *p* < 0.001) and infections (32% vs. 41%; *p* = 0.001) after study entry. In the ARDS network RCT, rapid discontinuation of study drug after extubation was associated with return to mechanical ventilation for 26% of methylprednisolone-treated patients from reconstituted systemic inflammation in the presence of adrenal suppression [19]. Despite the partial loss of early benefits associated with return to mechanical ventilation, patients randomized prior to day 14 had a 25% [18 of 66 (27.3%) vs. 24 of 66 (36.4%); RR 0.75, CI 0.45 to 1.24] and 31% [18 of 66 (27.3%) vs. 26 of 66 (39.4%); RR 0.69, CI 0.42 to 1.13] relative reduction in 60-day and 180-day mortality, respectively [19].

**Fig. 2:**
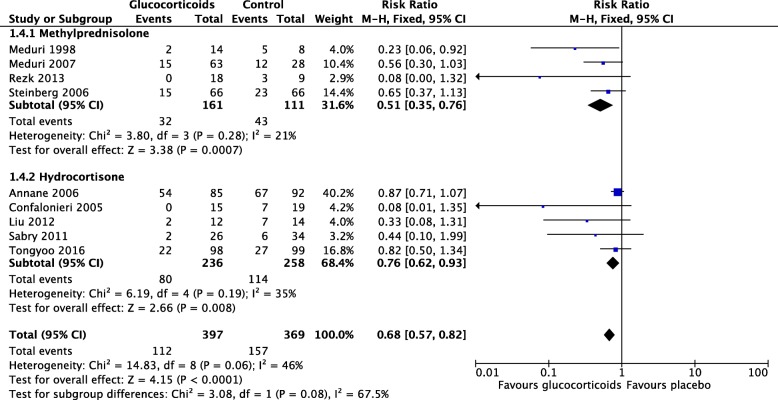
Forest plot of mortality in randomized trials of patients with ARDS, by glucocorticoid molecule. Hospital mortality for patients (*n* = 766) randomized before day 14 of ARDS onset in nine randomized trials investigating prolonged glucocorticoid treatment in ARDS. Comparison between randomized trials which investigated methylprednisolone (*n* = 272) vs. hydrocortisone (*n* = 494) treatment. M–H Mantel–Haenszel statistics, df degrees of freedom

During the consensus process, an additional RCT was published by Tongyoo et al. [20] (hydrocortisone 7 days, no bolus, no tapering) and incorporated in the final analysis of the nine trials (*n* = 816). Overall, glucocorticoid treatment was associated with reduction in MV-free days (mean difference 6.36 days, 95% CI 2.94–9.77; *p* < 0.001) and decreased hospital mortality for those randomized before day 14 of ARDS (Fig. 2, 28.2% vs. 42.5%, risk ratio 0.68, 95% CI 0.57–0.82, *I*^2^ 46%, *p* < 0.0001). The number to treat to save one life was 7. Except for transient hyperglycemia (mostly within the 36 h following an initial bolus), prolonged glucocorticoid treatment was not associated with increased risk for neuromuscular weakness [21], gastrointestinal bleeding, or nosocomial infections [9]. Importantly, the survival benefit observed during hospitalization persisted after hospital discharge with follow-up observations extending up to 1 year (limit of measurement) [3, 4, 16].

The task force suggests administering (Table 1) methylprednisolone in patients with early (up to day 7 of onset; PaO2/FiO2 of ≤ 200) moderate-to-severe ARDS in a dose of 1 mg/kg/day (ideal body weight) and late (after day 6 of onset) persistent ARDS in a dose of 2 mg/kg/day followed by slow tapering over 13 days (conditional recommendation based on moderate quality of evidence). Furthermore, the task force suggested that methylprednisolone should be weaned slowly (6–14 days) and not stopped rapidly (2–4 days) or abruptly as deterioration may occur from the development of a reconstituted inflammatory response [9]. Since glucocorticoid treatment blunts the febrile response, infection surveillance was recommended to promptly identify and treat hospital-acquired infections [9].

**Table 1:** Methylprednisolone treatment of early moderate-to-severe ARDS and late unresolving ARDS

Early moderate-to-severe ARDS (PaO_2_:FiO_2_ ≤ 200 on PEEP 5 cmH_2_0)		
Time	Intravenous administration form	Dosage
Loading	Bolus over 30 min	1 mg/kg
Days 1 to 14*^,†,‡^	Infusion at 10 cc/hour	1 mg/kg/day
Days 15 to 21*^,‡^	Infusion at 10 cc/hour	0.5 mg/kg/day
Days 22 to 25*^,‡^	Infusion at 10 cc/hour	0.25 mg/kg/day
Days 26 to 28*^,‡^	Infusion at 10 cc/hour	0.125 mg/kg/day
Unresolving ARDS = less than (a) one-point reduction in lung injury score or (b) or 100 improvement of in PaO_2_:FiO_2_ • By day 7 of ARDS in patients not receiving methylprednisolone for early ARDS • By days 5–7 of ARDS in patients receiving methylprednisolone (above) for early ARDS		
Time	Intravenous administration form	Dosage
Loading	Bolus over 30 min	2 mg/kg
Days 1 to 14*^,†,‡^	Infusion at 10 cc/hour	2 mg/kg/day
Days 15 to 21*^,‡^	Infusion at 10 cc/hour	1 mg/kg/day
Days 22 to 25*^,‡^	Infusion at 10 cc/hour	0.5 mg/kg/day
Days 26 to 28*^,‡^	Infusion at 10 cc/hour	0.25 mg/kg/day
Days 29 to 28*^,‡^	Bolus over 30 min	0.125 mg/kg/day

IV = intravenous. The dosage is adjusted to ideal body weight and round up to the nearest 10 mg (i.e., 77 mg round up to 80 mg). The bolus is given over 30 min. The infusion is obtained by adding the daily dosage to 240 cc of normal saline and run at 10 cc/hour

*Five days after the patient can ingest medications, methylprednisolone is administered per os in one single daily equivalent dose. Enteral absorption of methylprednisolone is compromised for days after extubation. Prednisone (available in 1-mg, 5-mg, 10-mg, and 20-mg strengths) can be used in place of methylprednisolone

^†^If between days 1 to 14 the patient is extubated, the patient is advanced to day 15 of drug therapy and tapered according to schedule

^‡^When patients leave the intensive care unit, if they are still not tolerating enteral intake for at least 5 days, they should be given the dosage specified but divided into two doses and given every 12 h IV push until tolerating ingestion of medications by mouth

Methylprednisolone may have several advantages over hydrocortisone for the treatment of ARDS. Methylprednisolone has (i) greater affinity for the glucocorticoid receptor (GR) [22], (ii) high penetration in lung tissue with longer residence time [23], and high potency for both (iii) genomic (inhibitory activity of transcription factor nuclear factor-kB) [24] and (iv) non-genomic activity [25]. Bolus administration prior to infusion achieves prompt elevation in plasma levels to assure higher GR saturation in the (i) cytoplasm and on the (ii) cell membrane for genomic and non-genomic actions, respectively. Cytoplasmic GR reach maximal saturation with approximately 100 mg methylprednisolone equivalent [26]. Bolus-associated increase in plasma methylprednisolone level causes transient hyperglycemia that does not affect the outcome. The methylprednisolone dose of 1 mg/kg/day in early ARDS is similar to the one commonly used in other forms of interstitial lung diseases [27] and in the IPDMA was associated—in comparison to placebo—with a threefold increase in the rate extubation by day 28 (HR 3.48, 95% CI 2.07–5.85; *p* < 0.0001) [13]. In critically ill patients, reduction in duration of mechanical ventilation is associated with a significant improvement in long-term outcomes including mortality, functional status, and quality of life [28, 29].

Observational studies and controlled trials have investigated the impact of early initiation of glucocorticoid treatment on preventing progression of the temporal continuum of systemic inflammation in patients with, or at risk for, ARDS. In a large retrospective observation study, among patients admitted to the ICU with sepsis, preadmission oral glucocorticoid treatment was independently associated with a lower incidence of ARDS (35% vs. 42%; *p* = 0.008) [30]. Two prospective controlled studies found that the intra-operative intravenous administration of methylprednisolone (125 or 250 mg) reduced the incidence of post-surgical ALI ARDS in patients undergoing pneumonectomy (*N* = 72; 0% vs. 13.5%, *p* < 0.05) [31] and esophagectomy (*N* = 234; 1.3% vs. 9.3%; *p* = 0.04) [32]. In aggregate data from four RCTs in patients (*n* = 945) hospitalized with community-acquired pneumonia, early prolonged glucocorticoid treatment prevented progression to ARDS (*N* = 945; 0.4% vs. 3.0%; RR 0.24, 95% CI 0.24, 0.10–0.56) [33]. In patients with early ARDS, prolonged methylprednisolone treatment prevented progression to respiratory failure requiring mechanical ventilation (42% vs. 100%; *p* = 0.02) [34] or progression to unresolving ARDS (8% vs. 36%; *p* = 0.002) [3].

Finally, the ARDS network RCT “Efficacy of Corticosteroids as Rescue Therapy for the Late Phase of Acute Respiratory Distress Syndrome (LaSRS)” [5] is frequently quoted—in isolation of the updated literature—to negate a therapeutic benefit for prolonged glucocorticoid treatment in ARDS [35]. Contrary to the misinformation associated with this publication, a recent re-analysis of the data demonstrates that methylprednisolone treatment was safe and highly effective in achieving disease resolution with sizable and significant improvements in all pre-specified secondary outcomes [19].

## Conclusions

In summary, there is moderate-to-high quality evidence that prolonged glucocorticoid therapy is safe and reduces (i) time to endotracheal extubation, (ii) duration of hospitalization, and (iii) mortality (number to treat to save one life = 7), and increases the number of days free from (i) mechanical ventilation, (ii) intensive care unit stay, and (iii) hospitalization. The mortality benefits—in comparison to placebo—persist after hospitalization. The adverse effects from glucocorticoids appear to be minimal and not important to patients (e.g., hyperglycemia). We believe that based on this evidence, all or almost all fully informed patients with ARDS would choose to use glucocorticoid therapy following the protocol outlined in Table 1.

## Abbreviations

ARDS: Acute respiratory distress syndrome
CIRCI: Critical Illness Related Corticosteroid Insufficiency
CPGD: Clinical practice guideline development
IPDMA: Individual patient data meta-analysis
IV: Intravenous
RCT: Randomized controlled trial
