# Supplementary material for: Prolonged low-dose methylprednisolone treatment is highly effective in reducing duration of mechanical ventilation and mortality in patients with ARDS
Source: J Intensive Care. 2018 Aug 24;6:53. doi: 10.1186/s40560-018-0321-9 (PMC6109298; doi:10.1186/s40560-018-0321-9)
Supplement: Related texts

# Reply: Response to Meduri et al.: “Prolonged low-dose methylprednisolone treatment is highly effective in reducing duration of mechanical ventilation and mortality in patients with ARDS”

**Authors:** Satoru Hashimoto, Masamitsu Sanui, Moritoki Egi

**Affiliations:** 6 0000 0001 0667 4960 grid.272458.e Department of Anesthesiology and Intensive Care, Kyoto Prefectural University of Medicine, Kyoto, Japan; 7 0000 0004 0467 0255 grid.415020.2 Department of Anesthesiology and Critical Care Medicine, Jichi Medical University Saitama Medical Center, Saitama, Japan; 8 0000 0004 0596 6533 grid.411102.7 Department of anesthesiology, Kobe University Hospital, Kobe, Japan

We read the letter to the editors from Dr. Meduri et al. with great interest. First of all, we congratulate their effort to elucidate the effects of prolonged treatment with low-dose methylprednisolone treatment for patients with ARDS. Guideline by Annane et al. clearly showed the beneficial effects of corticosteroids on ARDS [9]. Whereas our analyses for the guideline did not show any statistically significant reduction in mortality, a lower risk ratio in patients with glucocorticoids was suggested (0.83 [95% confidential interval 0.65, 1.07]) [1]. At least, the use of glucocorticoids may have less chance to increase the mortality risk, while the use of glucocorticoids was significantly associated with the longer ventilator-free days (mean difference [CI] 5.67 [3.49–7.68] days). Based on these results, our guideline suggested the administration of corticosteroids (equivalent to methylprednisolone 1–2 mg/kg/day) to adult patients with ARDS (GRADE 2B, “weak recommendation”/quality of evidence “moderate”).

As suggested by Meduri et al., it might be better not to include the study by Bernard et al., since the doses and duration of methylprednisolone were exceptional (i.e., large doses of methylprednisolone on a single day) [2]. Of note, however, the effects of glucocorticoids on mortality and infection rates did not reach a significant difference even after excluding this study (mortality: *p* = 0.14, risk ratio = 0.76 [95% confidential interval 0.52, 1.10]; infection rate: *p* = 0.08, risk ratio = 0.80 [95% confidential interval 0.62, 1.03]). Also, there are four studies included in the meta-analysis by Meduri et al., but not included in our analysis [13]. Two of the four studies examined patients with community-acquired pneumonia [15, 17]: one was written in Chinese (only English or Japanese literature included in our meta-analysis) [18] and the last one was not included in our search engines (i.e., MEDLINE, EMBASE, and Cochrane CENTRAL) [14]. One more study by Tongyoo cited in this study showed significant improvement in pulmonary physiology, but the study itself did not show significant survival benefit [20]. Despite an exclusion of these five studies, the results of our analysis are in the same line of their meta-analysis. And we agree with Dr. Meduri’s suggestion that methylprednisolone may have several advantages over hydrocortisone for the treatment of ARDS.

The very latest multicenter randomized controlled trial by Venkatesh et al. (ADRENAL trial) evaluated the effectiveness of low-dose hydrocortisone (at a dose of 200 mg per day) during the initial 7 days for 3658 mechanically ventilated patients with septic shock [36]. In this trial, the use of hydrocortisone did not decrease the 90-day mortality (*p* = 0.50), while patients with hydrocortisone had faster resolutions of shock than those with placebo (hazard ratio, 1.32; *p* < 0.001). However, there were no significant differences in the number of days alive and free from mechanical ventilation. Based on the results from ADRENAL trial and moderate quality of evidences noted in the meta-analyses for ARDS, more solid evidences are needed to recommend the routine use of glucocorticoids for patients with ARDS.

As Meduri et al. pointed out, large doses of methylprednisolone were occasionally administered for patients with ARDS or interstitial pneumonia in Japan. A survey for 296 hospitals in Japan revealed that so-called steroid pulse therapy (1000 mg of methylprednisolone for 3 days) was routinely used for patients with ARDS in approximately one third of the surveyed hospitals [37]. These unproven therapies should also be evaluated in future trials.
